# Involvement of inflammation and its related microRNAs in hepatocellular carcinoma

**DOI:** 10.18632/oncotarget.13530

**Published:** 2016-11-23

**Authors:** Ke Jin, Tong Li, Gonzalo Sánchez-Duffhues, Fangfang Zhou, Long Zhang

**Affiliations:** ^1^ Life Sciences Institute and Innovation Center for Cell Signaling Network, Zhejiang University, Hangzhou 310058, PR China; ^2^ Institutes of Biology and Medical Science, Soochow University, Suzhou 215123, PR China; ^3^ Department of Molecular Cell Biology, Cancer Genomics Centre and Centre for Biomedical Genetics, Leiden University Medical Center, 2300 RC Leiden, The Netherlands; ^4^ Institute of Pharmacology and Toxicology, College of Pharmaceutical Sciences, Zhejiang University, Hangzhou 310058, PR China

**Keywords:** inflammation, epithelial-mesenchymal transition, cancer stem cells, cell signaling, microRNAs

## Abstract

Hepatocellular carcinoma (HCC) is the fifth most commonly diagnosed type of cancer. The tumor inflammatory microenvironment regulates almost every step towards liver tumorigenesis and subsequent progression, and regulation of the inflammation-related signaling pathways, cytokines, chemokines and non-coding RNAs influences the proliferation, migration and metastasis of liver tumor cells. Inflammation fine-tunes the cancer microenvironment to favor epithelial-mesenchymal transition, in which cancer stem cells maintain tumorigenic potential. Emerging evidence points to inflammation-related microRNAs as crucial molecules to integrate the complex cellular and molecular crosstalk during HCC progression. Thus understanding the mechanisms by which inflammation regulates microRNAs might provide novel and admissible strategies for preventing, diagnosing and treating HCC. In this review, we will update three hypotheses of hepatocarcinogenesis and elaborate the most predominant inflammation signaling pathways, i.e. IL-6/STAT3 and NF-κB. We also try to summarize the crucial tumor-promoting and tumor-suppressing microRNAs and detail how they regulate HCC initiation and progression and collaborate with other critical modulators in this review.

## INTRODUCTION

Resolving inflammation is a component of the body's immune responses to external or internal stimuli that eliminates the aggressor and restores the tissue physiology. In contrast to resolving inflammation, non-resolving inflammation is a major driver of disease. It becomes clear that perpetuation of inflammation may lead to an inherent health risk, as the chronic inflammation can progressively damage the tissues [1]. Clinical and epidemiological studies have suggested that about 15% of human cancers are associated with chronic infection and inflammation [2]. Hepatocellular carcinoma (HCC) is the fifth most commonly diagnosed type of cancer [3]. Inflammation is central to the pathogenesis of chronic liver injury and has been proposed as a risk factor for HCC. In 2011, 8% of the world's population was chronically infected with hepatitis B or C viruses (HBV or HCV), thus increasing the risk of HCC development [4]. Up to 5% of HCV patients will develop HCC in their life-span [5].

The current understandings which concern HCC initiation and progression involve the epithelial-mesenchymal transition (EMT) [6–8], cancer stem cells (CSCs) [3, 9, 10] and inflammatory microenvironment hypotheses [11, 12]. As known, HCC usually progresses through four stages: cell degeneration, fibrosis, cirrhosis and tumor formation. Noteworthy, inflammation is involved in all of the stages [7]. During HCC initiation, cells acquire mutations that lead to inactivation of tumor suppressor genes and/or activation of oncogenes, thereby providing mutant cells with a growth and survival advantage [13]. However, these initial genetic or epigenetic changes are not sufficient for a complete neoplastic progression, suggesting that tumor promotion and progression might depend on consistently supportive signals that are likely to be released from tumor inflammatory microenvironment.

In this review, we will describe the underlying mechanisms driving liver tumorigenesis and the involvement of key inflammation related signaling pathways and microRNAs (miRNAs), which might facilitate to hypothesize about the etiology of HCC, as well as to develop the diagnosis and therapies for hepatocarcinogenesis.

## INFLAMMATION-RELATED EMT

EMT is involved in several steps of HCC initiation and progression. On one hand, hepatic stellate cells (HSCs), hepatocytes and some other cells undergo EMT in response to liver injuries and inflammatory stimuli to promote deposition of extracellular matrix (ECM), leading to liver fibrosis; on the other hand, primary HCC cells undergo EMT to gain invasive, migratory and stem cell-like properties allowing them to disseminate and propagate at distant sites [7]. EMT is mediated by genetic and epigenetic modifications in the cells. For instance, EMT activates transcription factors such as Snail, Twist and ZEB, down-regulating intercellular junction proteins such as E-cadherin, CAR, claudins, occluding and ZO-1 and up-regulating mesenchymal-related proteins such as Vimentin, Fibronectin, and N-cadherin [6, 8]. Meanwhile, miRNAs such as miR-155 and miR-200c are also capable of regulating EMT and linking inflammation with HCC initiation, which will be discussed later. Noteworthy, EMT requires cells responsive to EMT-promoting stimuli and an extracellular microenvironment able to provide cytokines and growth factors capable of inducing EMT. Furthermore, a number of publications point out that the inflammatory microenvironment enriched in cytokines, chemokines and growth factors as the optimal microenvironment to induce EMT. Among them, the transforming growth factor-β (TGF-β) has been unveiled as the predominant regulator of EMT.

**Figure 1:**
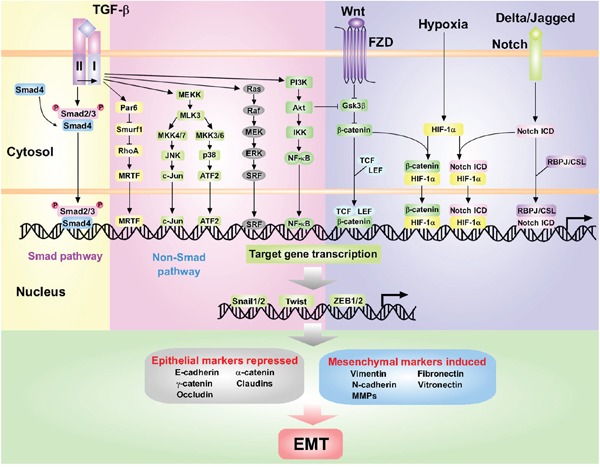
The dominating interconnected signaling pathways and transcriptional network that promote EMT during tumorigenesis TGF-β signaling pathway is initiated by binding of TGF-β ligands to TβRII and TβRI. The Smad pathway is mediated by phosphorylation of TβRI by TβRII and subsequent activation of Smad2/3. Activated Smad2/3 form complexes with Smad4 and translocate into the nucleus. The Non-Smad pathway takes place through multiple intracellular signaling cascades such as Par6-Smurf1-RhoA, RAS-RAF-MEK-ERK and PI3K/Akt pathway. Other signaling pathways, such as Wnt, Notch and HIF-1α, are also involved in EMT. Wnt signaling promotes EMT by inhibiting GSK3β to stabilize β-catenin, which translocates to the nucleus with LEF/TCF. The interaction between Delta/Jagged and its receptor Notch induces the release of Notch ICD. Hypoxia in the tumor microenvironment promotes EMT through HIF-1α and crosstalks with Wnt and Notch pathways. Activation of above pathways induces the expression of master regulators of EMT including Snail1/2, Twist and ZEB1/2 families, which can initiate a coordinated transcriptional network leading to suppression of epithelial marker and up-regulation of mesenchymal marker expressions.

The pleiotropic cytokine TGF-β exerts context-dependent actions on both EMT and hepatocarcinogenesis [14]. On one hand, TGF-β signaling may inhibit HCC development in an early stage of tumorigenesis; on the other hand, TGF-β supports the progression and maintenance of advanced HCC [15]. EMT can be activated through canonical or non-canonical manners downstream of TGF-β receptors (Figure 1). In the canonical pathway, TGF-β binds to the type I and type II Serine/Threonine kinase receptors (TβRI and TβRII) on the cell surface. The activated TβRI phosphorylates specific receptor-regulated R-Smad proteins, Smad2 and Smad3, which assemble into heteromeric complexes with Co-Smad4 [16, 17]. This pathway regulates the expression of EMT transcription factors including Snail, ZEB and Twist. In the non-canonical pathway, TGF-β stimulates various alternative signaling pathways such as mitogen-activated protein kinase (MAPK), small GTPases, phosphoinositide 3-kinase (PI3K)/Akt, and nuclear factor κB (NF-κB) to regulate expression of EMT-related genes (Figure 1). Under inflammatory microenvironment, TGF-β may accelerate the progression of EMT. For example, the aberrant TGF-β and interleukin-6 (IL-6) axis was reported to mediate selective and adaptive mechanisms of resistance to molecular targeted therapy in lung cancer [18]. In addition, a number of studies have uncovered several TGF-β-independent pathways involved in EMT activation in HCC and other cancers, including the recently reported role of phosphorylation of eukaryotic initiation factor-4E (eIF4E) to promote EMT and metastasis via translational control of Snail and matrix metalloproteinase (MMP)-3 [19]. Additionally, Hedgehog signaling-regulated hypoxia was demonstrated to induce EMT and invasion in pancreatic cancer cells [20]. Therefore, to interfere with EMT by intervening in the mechanisms by which the inflammatory microenvironment and TGF-β signaling cooperate, might be considered as a therapeutic approach for HCC.

## CANCER STEM CELLS AND INFLAMMATION NICHE

In the early steps of HCC progression, most of the tumor cells are sensitive to radiotherapy and chemotherapy. However, increasing evidences support the idea that a rare population of cells which exhibit self-renewal and tumorigenic potential called cancer stem cells (CSCs) are present in HCC, and perhaps all types of tumors [10, 21]. Actually, epidemiological data suggest that up to 40% HCCs develop from clonal populations originated from hepatic CSCs [22, 23]. Such cells are believed to be the only cells that exist in the liver for sufficient time to adopt the necessary genetic or epigenetic changes required for neoplastic development [24]. However, determination of the origin and spatio-temporal dynamics of liver CSCs remains to be accomplished. With this regard, hepatic tissue stem cells, or liver progenitor cells (LPCs), which are required to modulate liver development and homeostasis, share many similarities with liver CSCs [25]. Therefore one can speculate that LPCs can convert into CSCs under a certain tumorigenic microenvironment, especially the inflammatory microenvironment. In fact, a recent study showed that the malignant progression of LPCs might serve as a pre-malignant marker for HCC [5]. These LPCs are induced by diethylnitrosamine (DEN), a commonly accepted carcinogenic compound for liver, and the proliferation of LPCs in liver depends on autocrine IL-6 signaling [5]. LPCs derive from the *canal of hering* and subsequently differentiate into hepatocytes or cholangiocytes under certain conditions [26]. Upon injury, LPCs revive and expand from the *canal of hering* via a process called “Ductular Reaction” [27]. However, chronic liver injuries and associated regeneration may result in hepatocarcinogenesis. Therefore, identification of markers to characterize LPCs/CSCs in liver and study on signaling pathways associated with LPCs/CSCs fate determination, are critical to control HCC. Currently a number of markers, such as CD13, CD133, CD24, CD44, CD90, cytokeratin 19 (CK19), OV6, α-fetal protein (AFP) and epithelial cell adhesion molecule (EpCAM) [3, 5, 26, 28, 29] (also see Table 1) have been proposed to identify LPCs/CSCs. Yi Tang and colleagues reported that normal LPCs are characterized by the positive expression of octamer-binding transcription factor 4 (Oct4), signal transducer and activator of transcription 3 (STAT3), embryonic liver fodrin (ELF) and TβRII, whereas hepatic CSCs express Oct4 and STAT3 but lack ELF and TβRII [21, 30]. Meanwhile, signaling pathways that regulate the LPCs/CSCs fate have been suggested, such as bone morphogenetic protein (BMP), fibroblast growth factor (FGF), Wnt, oncostatin M (OSM), TGF-β, Jagged1/Notch, IL-6/STAT3, and hepatocyte growth factor (HGF)/c-Met [3, 31]. Moreover, expression patterns of miRNAs in EpCAM^+^ hepatic CSCs differ from that in LPCs, and miRNAs such as Let-7 family members, miR-125a/b and miR-452 are also involved in determination and maintenance of LPCs/CSCs [79–81]. It is worth noting that a series of intriguing questions have not been addressed. For example, HCC may arise from LPCs/CSCs, and chronic liver injury may cause HCC. As aforementioned, LPCs proliferate in response to liver injury to contribute to liver regeneration. Therefore, whether regenerating or tumorigenic cells respond to liver injury in different manners, for example by inducing either cell proliferation or apoptosis is still under debate. In this sense, one could benefit from making liver CSCs sensitive to death signaling under chronic liver injury.

**Table 1:** Putative biomarkers of liver CSCs

Biomarker	Location	Biological functions in liver CSCs	Characteristics of marker-positive CSCs (Sources)	Refs
ABCG2	Cell surface	Determinant of the SP phenotype; extruding a variety of compounds such as anticancer agents	Chemoresistant (PLC5, HepG2, Huh-7, MHCC-97L, Hep3B; Human HCC tissue)	[32–34]
AFP	Cytoplasm; secreted	Serum transport protein; binding numerous molecules (fatty acids, estrogen, steroids); modulating immune function, metabolism	Poorly differentiated, anti-apoptosis, cell cycle progression, tumorigenic, invasive, metastatic (Huh-1, HepG2, Hep3B, SK-Hep-1; Human HCC tissue; female athymic nude mice)	[35–38]
ALDH1	Cytoplasm	Catalyzing the oxidation of endogenous and exogenous aldehydes; functional marker of CSCs; cellular detoxification	Abnormal metabolism, chemoresistant, tumorigenic (H2P, H2M, Hep3B, QGY-7701, QGY-7703, BEL7402, HepG2, PLC8024, Huh-7; SCID mice)	[39, 40]
CD13	Cell surface	Reducing ROS-induced DNA damage; protecting cells from apoptosis	Tumorigenic, chemoresistant (Huh-7, PLC/PRF/5; Human HCC tissue; NOD/SCID mice)	[41–43]
CD24	Cell surface	Mediating Twist2/STAT3/Nanog self-renewal pathway	Tumorigenic, chemoresistant, metastatic (HLE, HepG2, MHCC-97L, MHCC-LM3, MHCC-97H, Huh-7, PLC/PRF/5, Hep3B, BEL7402; Human HCC tissue; NOD/SCID mice)	[44–46]
CD44	Cell surface	Reducing ROS level via stabilizing xCT; regulating TGF-β-mediated mesenchymal phenotype; mediating c-Met-PI3K-AKT signaling cascade	Tumorigenic, invasive, circulating (PLC/PRF/5, Huh-7, HLE, Huh-1, Hep3B, HepG2, SK-Hep-1, MHCC97-H, HLF; Human HCC tissue; Transgenic mice, Nude mice)	[47–51]
CD90	Cell surface	Involved in cell-cell, cell-matrix interactions	Tumorigenic, invasive, metastatic, circulating, chemoresistant, proliferation (Hep3B, MHCC-97L/H, Huh-7, SMMC7721, SK-Hep-1, PLC/PRF/5; NOD/SCID mice; Human HCC tissue)	[52–54]
CD133	Cell surface	Supporting tumor growth and survival; mediating Akt/PKB pathway and Neurotensin/Interleukin-8/CXCL1 signaling	Tumorigenic, chemoresistant (Hep3B, Huh-7, PLC8024, HepG2, SK-Hep-1; Human HCC tissue; SCID mice)	[28, 55–58]
CK19	Cytoplasm	Skeleton protein	Tumorigenic, invasive, metastatic, chemoresistant (Huh-7, PLC/PRF/5, Hep3B; Human HCC tissue; NOD/SCID mice)	[59–61]
DCLK1	Whole cell	Catalyzing tubulin polymerization into microtubules; regulating HCV replication	Tumorigenic, invasive, metastatic (Huh-7; Athymic nude Balb/c mice; Human HCC tissue)	[62, 63]
DLK1	Cell surface	Not reported	Tumorigenic, chemoresistant (PLC/PRF/5, QGY7701, SK-Hep-1, YY-8103, SMMC7721, HepG2, Hep3B, Huh-7, SNU398, WRL68, MHCC-97L, MHCC-LM3; NOD/SCID mice)	[64]
EpCAM	Cell surface	Cell-cell adhesion; maintenance of a pluripotent state; regulation of differentiation, migration and invasion	Tumorigenic, invasive, chemoresistant, circulating (Huh-7, Huh-1, Hep3B, PLC/PRF/5, SK-Hep-1, HLE, HLF; Human HCC tissue)	[37, 38, 52, 65–67]
KIAA1114	Cell surface	Not reported	Tumorigenic, metastatic (Hep3B, SK-Hep-1, Huh-7, HepG2, SH-J1, SNU475; Beige/nude/XID mice; Human HCC tissue)	[68]
Lin28B	Nucleus (main)	Regulating the transition between pluripotency and committed cell lineages	Metastatic, poorly differentiated, circulating (PLC/PRF/5, Huh-7, HepG2; Human HCC tissue; Transgenic mice)	[69–71]
OV6	Cell surface	Not reported	Tumorigenic, chemoresistant, invasive, metastatic (Huh-7, SMMC7721, HepG2, PLC/PRF/5, Hep3B; Human HCC tissue; NOD/SCID mice)	[72–74]
SALL4	Intracellular	Regulating embryogenesis, organogenesis, pluripotency	Cell cycle progression, chemoresistant (Huh-7, PLC/PRF/5; NOD/SCID mice; Human HCC tissue)	[75, 76]
TLR4	Cell surface	Receptor for LPS; facilitating invasion and migration	Invasive, metastatic (SMMC7721, Huh-7; Human HCC tissue; BALB/c-nu/nu mice)	[77, 78]

Abbreviations: ABCG2, ATP-binding cassette G subfamily type 2 transporter; SP, side population; ALDH1, Aldehyde dehydrogenase 1; DCLK1, doublecortin-like kinase 1; SALL4, Sal-Like Protein.

## INFLAMMATORY MICROENVIRONMENT

**Figure 2:**
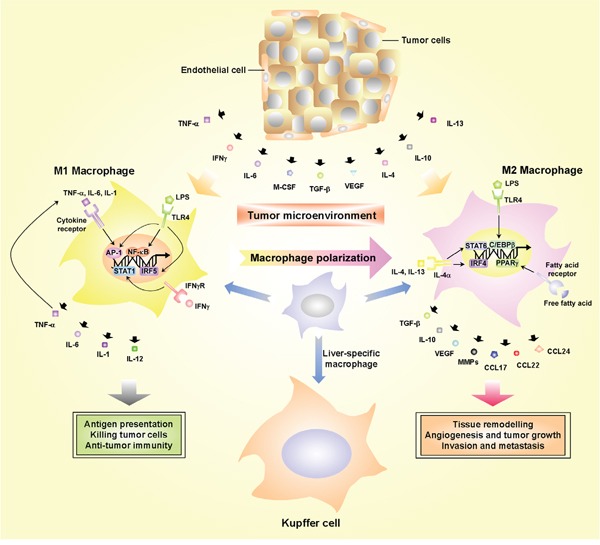
The roles of TAMs in the pro-inflammatory microenvironment Macrophages can be classified into two main classes according to their phenotypic polarization: M1 macrophages respond to IL-6, TNF-α, M-CSF, INFγ and LPS whereas they differentiate into M2 in response to TGF-β, VEGF, CCL2, IL-4, IL-10 and IL-13. M1 and M2 macrophages exert different functions. M1 macrophages with powerful antigen presentation potential can secrete IL-1, IL-6, IL-12 and TNF-α, and are able to exert cytotoxic activity on microbes and tumor cells. M2 macrophages can secrete VEGF, MMPs, IL-10 and TGF-β and promote angiogenesis, tissue remodeling, tumor progression, invasion and metastasis as well as suppression of anti-tumor immune response. TAMs can be recruited to tumor lesions and interact with both stromal and tumor cells within the tumor microenvironment, which will amplify the inflammation and accelerate tumor progression.

The inflammation-tumorigenesis cascade is probably orchestrated by various types of cells within the local inflammatory microenvironment and the pro- and anti-inflammatory molecules they produce. In the process of liver tumorigenesis, tumor-associated macrophages (TAMs) play a pivotal role between tumor cells and stromal cells [11, 82]. Macrophages derive from peripheral-blood monocytes and are recruited to the tumor sites by chemotactic factors such as chemokine CC motif ligand 2 (CCL2) and macrophage colony-stimulating factor (M-CSF). Macrophages can be broadly classified as M1 and M2 types according to their polarity (Figure 2). M1, with powerful antigen presentation potential, can rapidly respond to microbial products and interferon γ (IFN-γ). Then, they overexpress IL-12 and other pro-inflammatory factors that activate Th1 cytotoxic cells that target microbes and tumor cells by producing reactive oxygen species (ROS) and nitric oxide (NO) [83]. In contrast, monocytes differentiate to M2 when exposed to IL-4, IL-10, IL-13, the ligands of Toll-like receptor (TLR) and glucocorticoid. M2 are weak in antigen presentation potential and secrete IL-10, TGF-β and other chemokines such as CCL17, CCL22 and CCL24. M2 exert multiple functions, e.g., activating Th2 cells and promoting angiogenesis, tissue remodeling and recovery [12, 83]. TAMs within the tumor usually belong to the M2 type and clinical studies imply that increase of M2 type is related to angiogenesis, tumor metastasis and poor prognosis [12, 84]. Resident macrophages in liver, called Kupffer cells, appear essential for sensing liver injury and initiating inflammatory responses in HCC. For example, Xu and Tian reported that Kupffer cell-derived IL-10 plays a key role in maintaining humoral immune tolerance in HBV-persistent mice [85]. Moreover, recent observations in animal models revealed that hepatic macrophages are a remarkably heterogeneous population of immune cells that play diverse functions in homeostasis, disease progression, and regression from injury [86]. We can infer that besides Kupffer cells, other populations of immune cells such as dendritic cells, lymphocytes and natural killer cells may be involved in the initiation and progression of liver tumorigenesis as well.

Interestingly, liver tumorigenesis is also characterized by an abnormal secretion of pro-inflammatory cytokines, which further favor an inflammatory microenvironment. This is supported by a shift from the Th1 cell related cytokines (IL-4, IL-8, IL-10 and IL-5) to Th2 cell related cytokines (IL-1, IL-2 and tumor necrosis factor α[TNF-α]) [87, 88]. Other studies found that expression patterns of inflammatory cytokines differ among HCC cell lines. For example, in HepG2 cells, IL-1, IL-2, IL-4, IL-5, IL-6 and IL-8 expression are significantly lower than those in Huh7 cells [89]. In addition, inflammation-related chemokines and their receptors contribute to the pathogenesis of HCC in different aspects, such as promoting proliferation of cancer cells, fine-tuning the inflammatory microenvironment in the tumor, favoring evasion from immune surveillance and inducing angiogenesis and tumor metastasis [90]. Therefore, to block immune response in the liver such as using neutralizing antibodies to neutralize inflammatory cytokines or their receptors might be considered as a promising strategy to dampen liver tumorigenesis.

## INFLAMMATION-RELATED SIGNALING PATHWAYS

### IL-6/STAT3 signaling pathway

**Figure 3:**
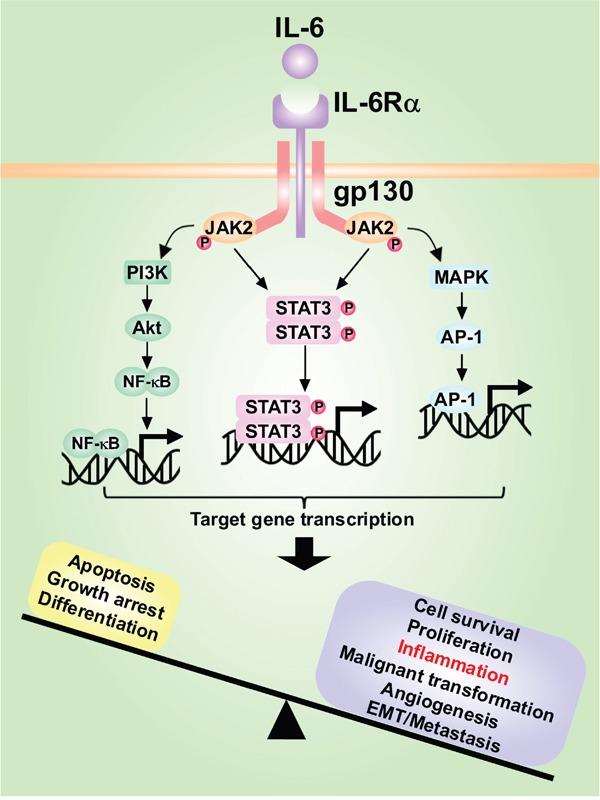
The role of IL-6/STAT3 signaling pathway and interactions with other pathways in hepatocarcinogenesis IL-6 secreted by Kupffer cells or hepatocytes binds to IL-6Rα and induces the homodimerization of IL-6Rα with gp130, activating downstream signaling pathways such as JAK/STAT3, PI3K/Akt and MAPK pathways, which promote proliferation and survival of cells, inflammatory amplification and tumor invasion and metastasis.

IL-6 plays a pivotal role in regulating multiple physiological and pathologic processes. For example, IL-6 is one of the early cytokines secreted during an acute phase of inflammatory response to promote liver regeneration, partly by up-regulating the expression of the fibrinogenic genes in the liver [86, 91]. IL-6 also induces insulin resistance in liver by activating STAT3 and stimulating the transcription of its target gene *suppressors of cytokine signaling 3* (*SOCS3*) [92]. In addition, IL-6 secretion by stromal cells has been reported to induce the formation of cancer stem-like cells [93], in a mechanism involving the up-regulation of Oct4 [94]. Furthermore, a positive feedback loop between IL-6 and NF-κB was elucidated in the recent years [95]. Among STAT members, the most studied is STAT3 which is closely associated with HCC. Upon IL-6 stimulation, the JAK/STAT3 signal transduction cascade becomes activated, which leads to phospho-STAT3 translocation into the nucleus and trans-activation of its target genes, including *protein inhibitors of activated STATs* (*PIAS*), *SOCS* and *SH2-containing phosphatases* (*SHP*), which are implicated in cell growth, proliferation, differentiation and survival [96]. Furthermore, STAT3 regulates many genes directly involved in the progression of HCC: (1) inflammation related genes: *IL-6*, *IFN*, *gp130*, *NF-κB*; (2) cell survival related genes: *Bcl-xL*, *Bcl-2*, *Survivin*, *XIAP*; (3) angiogenesis related genes: *vascular endothelial growth factor (VEGF)*, *FGF*, *platelet derived growth factor (PDGF)*; (4) cell proliferation related genes: *Cyclins*, *p21*; (5) tumor invasion and metastasis related genes: *cyclooxigenase (COX)-2/MMPs*; (6) oxidative stress related gene: *CYP450* [97]. Notably, chronic IL-6 stimulation was shown to induce tumorigenesis in liver [5, 98, 99], via a mechanism that might imply the over activation of STAT3 (Figure 3). Additionally, plenty of miRNAs can regulate or be regulated by IL-6/STAT3 signaling, which will be elucidated in detail later. As known, STAT3 signal is essential for sustaining pluripotency in embryonic stem cells, but seems redundant for mature cells. Particularly, over activation of STAT3 is sufficient to induce tumorigenesis. Therefore, a proper targeted therapy against STAT3 in tumor cells might be a strategy for tumor treatment, avoiding undesirable side effects which may affect physiological functions of mature cells.

### NF-κB signaling pathway

**Figure 4:**
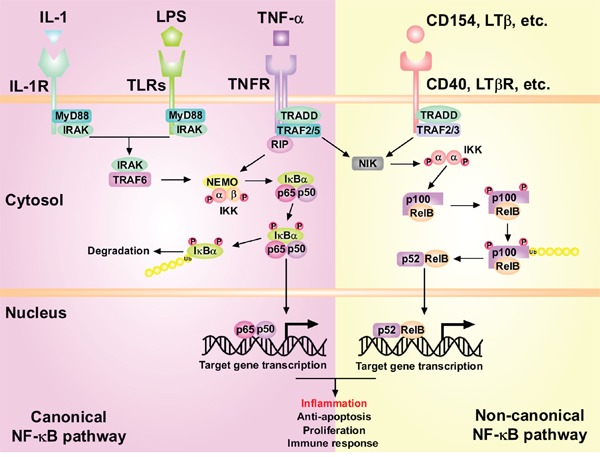
The activation of canonical and non-canonical NF-κB signaling pathways in the liver tumorigenesis In the canonical NF-κB pathway, IL-1, LPS or TNF-α activate IL-1R, TLRs and TNFR respectively, leading to the activation of the IKK complex which can phosphorylate IκB-α. This phosphorylation results in the polyubiquitination and subsequent proteasomal degradation of IκB-α. The released NF-κB p50-p65 dimers then translocate into nucleus and activate target gene transcription. In the non-canonical pathway, activation of CD40, LTβR, etc. leads to activation of IKK-α by NIK. IKK-α homodimers can then phosphorylate p100 subunit, which is a prerequisite for the polyubiquitination of p100 and its proteasomal processing to p52. Then RelB-p52 heterodimers translocate into nucleus and activate transcription of target genes.

NF-κB is probably the most studied signaling pathway in response to inflammation and refers to a family of signal-responsive transcription factors that, upon activation by exogenous ligands or cytokines such as lipopolysaccharide (LPS), TNF-α and IL-1, translocate into the nucleus and activate target genes [100]. NF-κB signals are regulated mainly by three components: (1) NF-κB family: Rel-A (p65), Rel-B, c-Rel, NF-κB1 (p105) and NF-κB2 (p100); (2) Inhibitor of κB (IκB) family: IκB-α, IκB-β, IκB-ε and Bcl-3; (3) IκB kinase (IKK) complex, including the catalytic subunits IKK-α, IKK-β, and the modulator IKK-γ (also termed as NEMO) [13]. P105 and p100 are processed to their mature forms p50 and p52, respectively [101]. The NF-κB pathway can be activated by canonical and non-canonical manners (Figure 4). In the canonical manner, activation of the IKK complex induces the phosphorylation and ubiquitin-mediated degradation of IκB-α, which releases the p65/p50 dimer that translocates into the nucleus. The non-canonical signaling involves the activation of the p52-Rel-B dimer derived from p100-Rel-B [102]. NF-κB modulates the transcription of various inflammatory cytokines and chemokines, functioning as a tumor-promoting regulator of HCC initiation [103]. NF-κB also interacts with many miRNAs to synergistically favor HCC development which will be discussed later. It is reported that IKK-β suppressed early chemical-induced liver tumorigenesis by inhibiting hepatocyte death and compensatory proliferation [104]. However, genetic inhibition of IKK-β long after tumor initiation accelerated HCC development and enhanced proliferation of tumor initiating cells, mainly by ROS accumulation and c-Jun N-terminal kinase (JNK) and STAT3 activation [104]. Although there are a large number of signaling pathways like TLR, TNF, TGF-β and Wnt that cooperate to modulate the promotion of HCC, many signaling pathways mentioned above seem to converge to the NF-κB pathway to exert their oncogenic effects, highlighting the crucial role of the NF-κB pathway.

### INFLAMMATION-RELATED MIRNAS

MiRNAs are endogenous single-stranded and evolutionarily conserved non-coding RNAs of 18-25 nucleotides in length [105]. They are mainly involved in the epigenetic regulation of gene expression at the post-transcriptional level by binding the 3′ untranslated regions (UTRs) of targeted messenger RNAs (mRNAs), resulting in the degradation of the targeted mRNAs and subsequent translational repression [106, 107]. MiRNAs have been demonstrated to regulate a myriad of physiological and pathological processes. Dysregulation of their expression has been linked to hepatic inflammation and tumorigenesis. All together, miRNAs establish complex regulatory networks involving transcription factors in order to promote or inhibit inflammation and carcinogenesis. The globally altered miRNAs in HCC have been reviewed elsewhere [108–111]. Therefore, next we will focus on miRNAs related to liver inflammation and tumorigenesis, and validate miRNA regulatory networks on HCC initiation and progression.

### Tumor suppressive miRNAs

#### miR-122

Genome-wide screening of miRNA expression alterations found that miR-122, a tissue-specific miRNA accounting for 70% and 52% of the total liver miRNAs in adult mouse and human respectively, is significantly decreased in the clinical HCC tissues and pre-clinical experimental studies [112–115]. MiR-122 contributes to maintaining the homeostasis of hepatocyte differentiation, cholesterol and fatty acid synthesis and metabolism in the healthy liver [113, 116]. Microarray analysis has shown that down-regulation of miR-122 promotes the dedifferentiation of hepatocytes [117]. Some targets of miR-122 in HCC have been elucidated, including *pyruvate kinase M2 (PKM2)*, *cut like homeobox 1 (CUTL1)*, *Ras homolog gene family member A (RhoA)*, *a disintegrin and metalloproteinase domain-containing protein (ADAM)-10 and -17*, *Cyclin G1*, *insulin-like growth factor 1 receptor (IGF1R)*, *Bcl-w*, *Wnt1* and *c-Myc*, which have been implicated in proliferation, apoptosis and metastasis of HCC cells [108, 116, 118, 119]. Hepatocyte nuclear factor (HNF)-1α, HNF-3β, HNF-4α, HNF-6 and CCAAT/enhancer binding protein α (C/EBPα) induce the expression of miR-122 [113, 118, 120]. MiR-122 also targets *Krueppel-like factor 6 (KLF6)*, a pro-fibrogenic factor, and miR-122-deficient mice developed hepatic inflammation, fibrosis and HCC, suggesting an anti-inflammatory role of miR-122 in the liver [114].

During HCV infection, miR-122 is essential for HCV replication, whereas appears to restrict HBV replication [120]. As aforementioned, 8% of the world population is chronically infected with HBV or HCV and up to 5% of HCV patients will develop HCC in their life [4, 5]. In this sense, whether miR-122 facilitates HCV replication and proliferation thus favoring HCC development or functions as a tumor suppressor simultaneously is unclear. As such, the expression levels of miR-122 that effectively promote HCV replication are undetermined. Moreover, whether the pathological microenvironment modulates the dual role of miR-122 remains unknown. These questions need to be solved to unveil the molecular mechanisms driving inflammation-related liver tumorigenesis.

#### miR-124

MiR-124 was initially identified as a brain-specific miRNA regulating neural development, inhibiting the proliferation of glioblastoma multiforme cells and inducing the differentiation of brain tumor stem cells [121, 122]. More recently, several publications have studied the roles of miR-124 in HCC, and a few targets of miR-124 have been confirmed, such as *Rho-kinase 2 (ROCK2)*, *an enhancer of the zeste homologue 2 (EZH2)*, *SET and MYND domain containing 3 (SMYD3)*, *STAT3* and *phosphoinositide 3-kinase catalytic subunit α (PIK3CA)*, through which miR-124 exerts its tumor-suppressive function [123–126].

**Figure 5:**
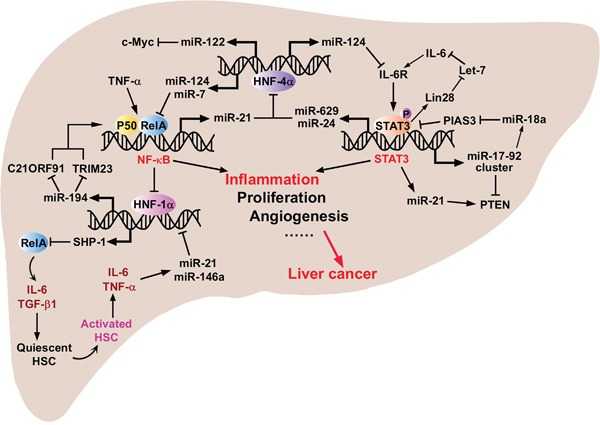
The critical crosstalks between important transcriptional factors, oncogenic and tumor suppressive proteins, and inflammation-related miRNAs that regulate key processes during HCC initiation, progression and metastasis The core associated proteins and miRNAs can constitute positive or negative feedback circuits to sustain the malignant state when there is an exogenous stimulus triggering the malignant transformation, and even when the stimulus is removed.

Furthermore, a novel role of miR-124 has been unveiled in two recent reports, describing previously unknown inflammatory feedback circuits involving miRNAs and transcription factors that amplify tumorigenic signals in HCC (Figure 5). First, it was shown that transient inhibition of HNF-4α is sufficient to initiate malignant transformation through a network including HNF-4α, miR-124, IL6R, STAT3, miR-24 and miR-629 [127]; the second pathway is comprised of HNF-4α, miR-124, miR-7, NF-κB (p65) and miR-21, which modulates HCC initiation and progression, and might be useful to predict the prognosis of HCC patients [128]. Interestingly, either a transient inhibition or activation of any component in the aforementioned pathways is sufficient to induce HCC initiation. Further, stable transformation can be supported by these feedback loops through multiple generations of cells, even if the initial stimuli are removed [129]. Particularly, miR-124 (alone or together with miR-7) inhibits the activation of IL-6R and NF-κB (Rel-A) and could be down-regulated by loss of HNF-4α. These results underline the key contributions of miRNAs to early stages of hepatocarcinogenesis.

#### miR-194 and miR-370

Previous investigations have identified the role of miR-194 and miR-370 in the lipid metabolism, liver fibrosis and HCV infection [130–133]. Chunyang Bao and colleagues delineated a network activated upon TNF-α stimulation, involving NF-κB, HNF-1α, miR-194, tripartite motif containing 23 (TRIM23), and chromosome 21 open reading frame 91 (C21ORF91) [134] (Figure 5). TNF-α-induced activation of NF-κB and inhibition of HNF-1α led to down-regulation of miR-194. *TRIM23*, which encodes an E3 ligase for NEMO ubiquitin conjugation and NF-κB activation [135], and *C21ORF91*, a gene of unknown function, are identified as direct targets of miR-194 in HCC cells. Upon knockdown of miR-194, its repressive effect on *TRIM23* and *C21ORF91* is relieved, rendering the activation of NF-κB and promoting HCC cell migration, invasion, and tissue colonization [134].

Another regulatory circuit described by Wen-Ping Xu and colleagues consists of miR-370, LIN28A, NF-κB (RelA/p65) and IL-6 [136]. The role of miR-370 in tumorigenesis remains controversial. Whereas evidence showed that miR-370 serves as a tumor suppressor in malignant cholangiocytes, leukemia cells and oral squamous carcinoma cells [137–139], several studies have reported that overexpression of miR-370 contributes to the progression of gastric carcinoma, prostate cancer, and acute myeloid leukemia [140–142]. In this report, miR-370 is down-regulated in HCC tissues and cell lines. They demonstrated that *LIN28A* is a direct target of miR-370, and blocks maturation of miR-370 in turn, forming a reciprocally repressive regulation [136]. Furthermore, they also verified that as an RNA-binding protein, LIN28A could directly bind to the RelA/p65 mRNA to promote its translation [136]. Finally, it was shown that IL-6 treatment on HCC cells significantly decreased miR-370 levels, which was followed by an increase in LIN28A protein, thereby closing the loop [136]. To our knowledge, let-7 is the only miRNA that interacts reciprocally with *Lin28*. NF-κB has been shown to transcriptionally activate the expression of LIN28B, rather than LIN28A, in breast cancer [136, 143]. This investigation not only elucidated the effect of LIN28A on NF-κB, but also identified a novel recripocal regulation between miR-370 and LIN28, thus updating our understanding of the interplay between miRNAs and RNA-binding proteins.

#### miR-15, miR-26, and miR-29 families

An increasing number of reports have unveiled a direct reciprocal regulation of these miRNAs with components of the NF-κB signaling pathway. For instance, miR-26b has been reported to suppress NF-κB signaling and enhance the chemosensitivity of HCC cells by inhibiting TGF-activated kinase 1 (TAK1) and TAK1-binding protein 3 (TAB3), two positive regulators mediating the activation of canonical NF-κB pathway [144]. Besides, NF-κB could promote the down-regulation of miR-29 in HSCs during liver fibrosis [145]. More importantly, Jie Ding and colleagues have identified the entire miRNA families or clusters that regulate almost all the steps in the NF-κB pathway, among which miR-195, a member of the miR-15 family, plays a crucial role in regulating the TNF-α/NF-κB pathway by down-regulating IκB-α and TAB3 in HCC [146]. It was previously mentioned that NF-κB plays a critical role linking inflammation to liver carcinogenesis, and miRNAs interacting with this pathway are also potential participants to involve the inflammation-related hepatocarcinogenesis.

### Oncogenic miRNAs

#### miR-155

As a multifunctional miRNA, physiological level of miR-155 has been shown to regulate the haematopoietic lineage differentiation and the homeostasis of the immune system [147, 148]. Recently, accumulating evidences have pointed out the oncogenic role of miR-155, which is frequently overexpressed in HCC. MiR-155 can be induced by a broad range of pro-inflammatory cytokines (e.g. TNF-α, IFN-γ, TGF-β) and activated by NF-κB and TLR ligands (e.g. LPS) [149], functioning as a critical link between inflammation and hepatocarcinogenesis. The total number of predicted potential targets of miR-155 (according to miRBase Sequence database) is 991 although only a limited number of these genes have been experimentally validated such as *C/EBPβ* and *SOCS1* [150].

MiR-155 expression can be transiently induced upon the activation of macrophages, dendritic cells, B cells and T cells, through the NF-κB and activator protein (AP)-1 [151]. Bo Wang and colleagues investigated the role of miR-155 at the early stage of non-alcoholic steatohepatitis-induced HCC and up-regulation of miR-155 accompanied by reduced expression of C/EBPβ and activation of NF-κB was confirmed in the liver during HCC progression [152]. Furthermore, deficiency of miR-155 attenuates liver steatosis and fibrosis in a mouse model of steatohepatitis without reducing inflammation [153]. In another study, miR-155 levels were markedly increased in patients chronically infected with HCV [154]. In addition, it was shown that miR-155 expression was up-regulated in non-parenchymal liver cells during HCV infection and that IL-10, TGF-β and miR-155 may regulate the TLR3-dependent antiviral and inflammatory activity of non-parenchymal liver cells *in vitro* [155]. Besides, miR-155 overexpression not only strongly enhanced the EMT process and cell invasion but also increased the population of stem-like CSCs among liver cancer cells [156]. Furthermore, knockdown of miR-155 in Kupffer cells resulted in immunosuppressive effects and prolonged mice survival using a liver allografts model [157]. In contrast, another study demonstrated that miR-155 was down-regulated in hepatocytes during chronic HBV infection and overexpression of miR-155 could contribute to reducing HBV viral load by targeting *C/EBPβ* [158]. Since HBV and HCV infection usually induce chronic hepatic inflammation, thereby favoring HCC initiation and progression, additional studies concerning the function of miR-155 in HCC may be considered in the future.

#### miR-21

MiR-21 has been strongly associated with anti-inflammatory response in macrophages, apart from regulating organ morphogenesis during embryonic development [159–161]. Up-regulation of miR-21 has been observed in almost all types of cancers [159]. A number of genes have been found to be targeted by miR-21, such as *phosphatase and tensin homolog (PTEN)* [162], *programmed cell death 4 (PDCD4)* [163, 164], *tissue inhibitor of metalloproteinase 3 (TIMP3)* [164, 165] and *p53* [166].

Inflammatory stimuli such as pro-inflammatory cytokines or HBV/HCV infection can induce the expression of miR-21. For example, IL-6 activates miR-21 through direct binding of STAT3 to an upstream enhancer of *miR-21* [167–169]. Additionally, miR-21 has been described to be part of a regulatory network involving HNF-1α, SHP-1, NF-κB (p65), STAT3, miR-146a and miR-21, which modulates hepatic fibrogenesis [167] (Figure 5). More interestingly, a coordinated crosstalk between hepatocytes and HSCs participates in this circuit and facilitates the progression of hepatic damage [167]. Impaired hepatocytes release IL-6 and TGF-β1 to activate the quiescent HSCs and activated HSCs release IL-6 and TNF-α up-regulating miR-21 and miR-146a to further aggravate the hepatic damage. HBV or HCV infection can also induce miR-21, and in addition to promoting viral replication, miR-21 also modulates the host response in favor of the virus [163, 170]. In this case, signaling components of the TLR pathway (*MyD88* and *IRAK*) have emerged as targets for miR-21, which lead to decrease of IFN-α [170]. On the other hand, miR-21 is significantly up-regulated in HCC samples from patients infected with HBV, but not HCV, when compared to adjacent benign tissue [171]. This finding was confirmed by a later publication which showed that miR-21 was among the most highly overexpressed miRNAs in hepatitis B, positive cirrhotic liver and HCC biopsies compared to healthy liver [172]. From this point of view, more clinical and pre-clinical experiments need to be done to elucidate the specific roles of miR-21 in different aspects of HCC progression.

#### miR-224

The expression of miR-224 is undetectable in normal livers, however, as liver diseases progress, the level of miR-224 increases, and can be elevated by over 20-fold [108, 145]. Cecilia Scisciani and colleagues identified p65/NF-κB as a direct transcriptional regulator of miR-224 and linked miR-224 up-regulation with activation of LPS, lymphotoxin-α and TNF-α inflammatory pathways, as well as cell migration/invasion in HCC from HCV-infected patients [173]. A later study demonstrated that autophagy suppressed tumorigenesis of HBV-associated HCC through degradation of miR-224 and derepression of its target *Smad4* [174]. A significant correlation was found between poor survival rate upon HBV infection and high expression of miR-224 [174, 175]. In a different report, HBV-associated tumors and tumors from HBx-transgenic mice were shown to have increased levels of miR-224, and miR-224 is a direct target of HBx and modulates HBV replication [176]. Taken together, miR-224 plays an important role in affecting crucial processes during HCC promotion and emerges as a new link between inflammation and HCC.

#### miR-17-92 cluster

The polycistronic miR-17-92 cluster encodes six miRNAs (miR-17-5p, miR-18a, miR-19a, miR-19b, miR-20a, and miR-92a-1) and has two paralogs in the human genome, the miR-106b-25 cluster and the miR-106a-363 cluster [177]. The miR-17-92 cluster has pleiotropic functions during both normal development and malignant transformation [178]. All the members of the miR-17-92 cluster are often overexpressed in HCC [172]. A number of transcription factors directly regulating the expression of this cluster have been revealed such as c-Myc [179] and E2F1 [180]. Compelling evidences have demonstrated that IL-6 regulates the expression of the miR-17-92 cluster by direct binding of STAT3 to its promoter region [181–183]. This emphasizes a potential link between miR-17-92 cluster and inflammation. Typically, up-regulation of miR-17-92 cluster by IL-6/STAT3 promotes cholangiocarcinoma growth via repression of the downstream target *PTEN* [183] (Figure 5). In addition, chronic exposure of HepG2 cells or primary hepatocytes to inflammation stimulates the expression of the miR-17-92 cluster member miR-18a [182]. This miRNA targets *PIAS3*, leading to an enhancement of STAT3 activity that eventually results in the release of acute-phase proteins [182] (Figure 5). This represents a novel positive feedback loop of IL-6 signaling through the involvement of miRNAs. Furthermore, other studies have investigated the molecular basis for miR-17-92 cluster pleiotropic functions in a cell type- and context-dependent manner [177]. For instance, miR-19a and miR-19b have been demonstrated to inhibit HSC-mediated fibrogenesis, either by negatively regulating *TβRII* and *Smad3* [184] or by repressing the hepatic fibrogenic master switch *connective tissue growth factor (CTGF)* [185]. Moreover, induction of HBV replication in a human hepatoma cell line increased miR-17-5p, miR-20a and miR-92a-1 expression via c-Myc [179]. Since miR-20a and miR-92a-1 directly inhibit HBV replication, this mechanism exemplifies a negative feedback regulation between HBV and the miR-17-92 cluster [179]. In summary, additional work has to be done to elucidate the mechanisms by which members of miR-17-92 cluster regulate HCC initiation and progression.

### Circulating miRNAs

Aberrant expressions of circulating miRNAs have also been widely reported in liver inflammation and related HCC. Many of the above discussed miRNAs such as miR-122, miR-29, miR-155 and miR-21 can function as circulating miRNAs and serve as biomarkers of liver inflammation and related HCC. For instance, increased circulating miR-122 is reported to be associated with drug-induced liver injury (DILI), HCV infection and HCC [186, 187]. Higher plasma miR-21 level is a promising biochemical marker for HCC and superior to AFP when distinguishing HCC from chronic hepatitis [188]. Circulating let-7 levels in plasma and extracellular vesicles correlate with hepatic fibrosis progression in chronic hepatitis C, although a single determination of let-7 levels in plasma does not have superior predictive value for significant hepatic fibrosis compared to that of fibrosis-4 index [189]. As regulators of inflammation, circulating miR-155 and miR-146a can be transferred between dendritic cells in the spleen, liver and bone marrow within exosomes to regulate inflammatory gene expression and response [190]. Because HCC is a highly complicated and heterogeneous disease and numerous miRNAs are dysregulated during HCC onset and progression, multiple circulating miRNAs and/or a combination with some of the biomarkers summarized in the Table 1 rather than a single circulating miRNA may increase the specificity and sensitivity for HCC diagnosis and prognosis prediction.

**Figure 6:**
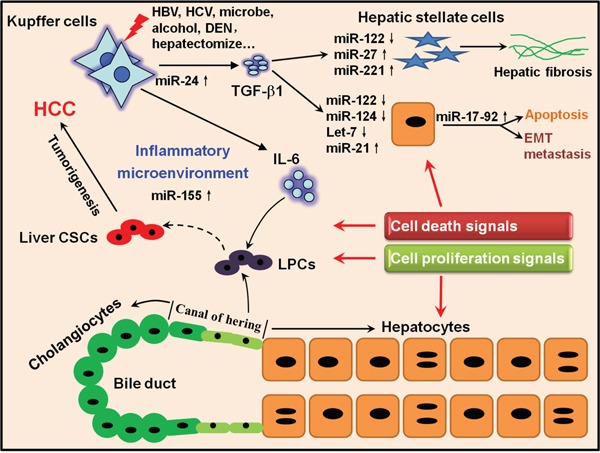
A hypothetical illustration delineating the connection between activation of inflammatory pathways, miRNAs and liver tumorigenesis Once extrinsic stimuli such as HBV/HCV, alcohol and DEN damage the liver, Kupffer cells can be activated and produce several inflammatory cytokines such as IL-6 and TGF-β1. On one hand, IL-6 can stimulate LPCs residing in the *canal of hering* to proliferate to restore the injured liver; however, if gene mutations happen to proliferating LPCs, they will have the potential to develop to CSCs. On the other hand, TGF-β1 can act on HSCs and activated HSCs proliferate and generate ECM to reconstitute the liver and promote hepatic fibrosis if the dynamic balance of ECM synthesis and decomposition is disrupted. Meanwhile, TGF-β1 can also stimulate hepatocytes to respond to either cell death or proliferation signals under different conditions. Several miRNAs such as miR-122, miR-155 and miR-21 could join to regulate correlated pathologic processes. All the cytokines, miRNAs and other inflammatory mediators together generate an inflammatory microenvironment which will amplify the oncogenic mutations and self-reinforce the pro-inflammatory signals, finally leading to the irreversible liver tumorigenesis.

In a word, the various roles of miRNAs linking inflammation to tumorigenesis during HCC progression are still largely unknown. Despite the recent discoveries of new signaling networks regulated by miRNAs in response to inflammation, it becomes evident that more dissections into the targets and regulators of miRNAs may contribute to improving our understanding of the molecular basis of liver tumorigenesis and thereby developing new approaches to treat HCC. A simplified illustration summarizing the proposed processes of hepatocarcinogenesis is shown as Figure 6.

## PROSPECT

Currently, the treatments of HCC consist of liver resection, transplantation, percutaneous ablation, chemoembolization, and systemic therapies [191]. Until 2007, no systemic chemotherapy was recommended for patients with advanced HCC [191, 192]. Sorafenib (Nexavar), a small multi-kinase inhibitor which targets VEGFR, PDGFR and Raf family kinases among others, was the first approved systemic therapy for HCC in 2007 and the only one that has been shown to significantly improve overall survival in patients with unresectable HCC [193]. In order to circumvent drug resistance, the combination of anticancer drugs has been proposed. The current evidence from the available clinical trials suggests that combined treatment of sorafenib with some anticancer agents (especially mTOR inhibitor) could be more effective for HCC [194]. Nevertheless, the development of additional treatment options with no or minor side effects, as well as identification of novel biomarkers of HCC that allow early detection, would significantly improve the prognosis of HCC patients. As aforementioned, a number of miRNAs have been associated with inflammation related HCC. It raises a question that whether “miRNA missile” could be a good strategy for HCC treatment, especially for those patients whose specific molecular disorder has been found. Theoretically, modulation of specific miRNA clusters might allow to reconfigure selected cancer cells to become less aggressive, and the tissue microenvironment to be less permissive to tumor progression. In this sense, miRNAs could achieve a selective activation of the immune system to contribute to eliminating cancer cells, without favoring their dissemination to distant organs. Finally, we have described how HCC results in changes in the expression of miRNAs. Therefore identification and profiling of circulating miRNAs linked to HCC might be helpful to favor an early detection of the disease.
